# The Australian Health Care Agreements 2003–2008

**DOI:** 10.1186/1743-8462-1-5

**Published:** 2004-11-18

**Authors:** Stephen J Duckett

**Affiliations:** 1 Professor of Health Policy La Trobe University Australia

## Abstract

The Australian Health Care Agreements for the five years 1 July 2003 to 30 June 2008 were signed in August 2003 after vituperative debate and intransigence from the Commonwealth that vitiated the negotiation process. The new Agreements, which were not as generous as the Agreements they replaced, increase accountability on the States, requiring States to match increases in Commonwealth funding, and de-emphasise the prospects for further reform in Commonwealth-State relations during the course of the Agreements. This paper describes the new Australian Health Care Agreements and the process which led to them.

## Introduction

The most significant Australian health policy event for 2003 was the signing of the five-year Australian Health Care Agreements. The Agreements were preceded by an ultimatum to the States and Territories from the Commonwealth indicating that there would be no changes from the offer on the table. This led to bitter political recriminations, but the Agreements were eventually signed.

In fact important preparations for Agreement renewal occurred in April 2002 with the Commonwealth and State Ministers, in a display of remarkable amity and accord, endorsing a new approach to the Agreements that:

• Commonwealth/State relations in the health arena should focus on the provision of optimal care and health outcomes, regardless of jurisdictional boundaries.

• It is in the best interests of all Australians for the Commonwealth, Stats and Territories to work co-operatively to improve the health and wellbeing of the community and the way in which health services are provided;

• The 2003–08 Agreements should contain the principles, objectives and proposed health outcomes designed to achieve those objections.

The Ministers also agreed to establish nine reference groups to address key issues in health reform which would feed into the Agreement "negotiation" process [1]. The reference groups addressed interaction between hospital funding and private health insurance; improving rural health; interface between aged and acute care; continuum between preventative, primary, chronic and acute models of care; improving indigenous health; improving mental health; information technology, research and e-health; quality and safety; and collaboration on workforce, training and education.

The reference groups were co-chaired by senior Government officials and non-Government clinical experts involving participants from the bureaucracies, people who work in health agencies, and consumers. The reference groups created great expectations amongst the neophyte health policy contributors who believed the rhetoric of the Commonwealth about being prepared to consider wide ranging changes to the health sector.

Seasoned commentators also called for reform [2-4]. Although extensive reports were produced by the Reference Groups and delivered to the Health Ministers the reports had no discernible impact on the 2003–2008 Agreements [5].

On 23 April 2003 the Commonwealth produced a non-negotiable offer with severe penalty clauses if States refused to sign by the Commonwealth's arbitrary deadline of 31 August 2003. An Australian Health Reform Alliance was formed to put pressure on the Commonwealth to respond to the reference group reports and to attempt to ensure that the 2003–2008 Agreements did not waste yet another opportunity to improve the efficiency, equity and quality of the health system. The Alliance's National Health Summit, which met at Old Parliament House, presented its final communiqué to non-Government politicians following a march up the hill to New Parliament House [6]. The Commonwealth deadlines remained and there was no change to the Agreement content.

The Commonwealth's confrontationalist stance effectively destroyed relationships between the Minister for Health and Ageing, Senator Kay Patterson, and her State colleagues, and she was replaced as Health Minister by Tony Abbott MP in the Ministerial reshuffle of October 2003.

## The content of the Agreements

There have been five Health Care Agreements since Medicare was introduced in 1984. The emphasis, orientation and priorities of these Agreements have changed over time (see Table 1).

**Table 1:** Key elements of Commonwealth-state hospital funding agreements

*Agreement*	*Political Objective*	*Key Principles*
1984–88 : Labor (Medicare Compensation Agreement)	Introducing Medicare	Compensation for cost increases and revenue losses
1988–93 : Labor (Medicare Agreement)	Consolidating Medicare Growth and reform of public provision	Incentives for system reform Penalties for lower public:private bed day shares and excess private medical service use
1993–98 : Labor (Medicare Agreement)	Entrenching Medicare Expansion of public provision	Reward for relatively higher levels of public provision and for increasing public provision relative to other states Post 1996, accountability for negotiated outcomes
1998–2003 : Coalition (Australian Health Care Agreement)	Continuing with Medicare Increased Commonwealth funding with increased accountability for states	Increased accountability on states for activity level changes Increased clarity of Commonwealth responsibility if health insurance levels change
2003–08 : Coalition (Australian Health Care Agreement)	Continuing with Medicare Slowed Commonwealth funding growth Increased accountability for states	Improved reporting, including of state spending Requirement on states at least to match Commonwealth funding increases

Source: [11]

The most significant elements of the 2003–08 Agreements are:

• a base grant which is increased for weighted population increases, a further 1.7 per cent increase for utilisation drift, and indexation for wage movements

• a withheld amount of 4 per cent of the grant paid on compliance with reporting schedules and funding growth matching requirements

• a capital funding scheme to facilitate improvements in services involved in the transition from hospital to home ('Pathways Home Program')

• funding for palliative care, mental health, and safety and quality initiatives.

The most contentious difference between the 1998–2003 and 2003–2008 Agreements related to the indexation provisions (see Table 2 for significant areas of difference between the two Agreements).

**Table 2:** Comparison of provisions of 1989–2003 and 2003–08 Australian Health Care Agreements

***Agreement Provision***	***1998–1998 Agreement***	***2003–2008 Agreement***
Indexation	2.1% above weighted population growth applied to 83% of the grant	1.7% above weighted population growth applied to 71% of the grant
State matching	Nil	State "commits to increase its own source funding for public hospital services such that the cumulative rate of growth will at least match the cumulative rate of growth of Commonwealth funding" (Clause 11)
Scope and level of services	(State) "continues to provide services to public patients at an indicative public patient weighted separation rate of XX" (Clause 22)	"The range of services available to public patients should be no less than was available on 1 July 1998" (Clause 7(a))
Reform	The Commonwealth and Victoria recognise the need for service delivery reform and ongoing exploration of additional initiatives under a measure and share model. Victoria will work with the Commonwealth in evaluating the outcomes from the Co-ordinated Care Trials to provide information to guide future directions for the reform of health service delivery. The Commonwealth and Victoria will consider proposals which move funding for specific services between Commonwealth and State funded programs on the basis that each proposal meets the following criteria: • the proposal must be consistent with accepted evidence based best practice care models; • there should be a sound basis for believing that the reform will lead to improved patient outcomes and/or more cost effective care; • the impact of the proposal should be measurable in terms of change in services delivered and costs to the health system as a whole and to each party to this Agreement; • if the proposal is expected to lead to net savings, these should be shared equitably between the Commonwealth and Victoria; • the proposal should have potential to be replicated, be on a scale such that extension can be realistically tested and be evaluated in terms of such extension; and • the proposal must preserve eligible persons' current access to Medicare Benefits Schedule services or their equivalent. Reform proposals may result in the cashing out of State funded programs and/or Commonwealth funded programs, including the Medicare Benefits Schedule and Pharmaceutical Benefits Scheme.	Victoria and the Commonwealth are committed to working with other States to progress the reform agenda agreed by Commonwealth and State Ministers for Health on 27 September 2002. The Commonwealth considers that for its part, such reform can taken place within existing funding parameters. In line with clause 18, the specific areas of national co-operation to deliver reform include: (a) improving the interface between hospitals and primary and aged care services; (b) achieving continuity between primary, community, acute, sub-acute, transition and aged care, whilst promoting consumer choice and improved responsiveness. Initial priorities for a stronger continuum of care approach will be cancer care and mental health services; and (c) exploring setting up a single national system for pharmaceuticals across all settings.

Each of the predecessor Agreements provided indexation formulae to account for growth and ageing of the population. The 1998–2003 Agreements also recognised that there was further "utilisation drift", that is increases in utilisation were occurring in the hospital sector over and above that which can be explained by population growth and ageing. This utilisation drift was in part the result of new technologies that allowed for treatments for conditions for which there was previously no hospital treatment. Utilisation also increased because of shifts in treatment from general practitioners' rooms and other ambulatory settings to same day hospital admission.

The 1998–2003 Agreements provided an escalation factor of 2.1% per annum over and above the growth caused by the increase in the rate of population for key elements of the grant. The 2003–08 Agreements reduced the utilisation drift factor to 1.7% and narrowed the applicable components of the grant, saving the Commonwealth Government about $1 billion from that provided for in the Forward Estimates. This reduction in growth provision was vociferously opposed by States and also by clinicians who were experiencing significant financial pressures on hospitals as a result of State Government funding constraints.

The 2003–08 Agreements also addressed an ongoing concern of Commonwealth Governments (both Labor and Coalition): its perception that when the Commonwealth increased expenditure on hospital services, this often had no discernible impact on hospitals as State Governments withdrew funding concomitantly. As Deeble points out, the reality is more complex, but the evidence is that an increased Commonwealth share is associated with growth in spending [7]. The new Agreements provided that the States were required to increase their funding of hospitals at the same rate as the Commonwealth increases, otherwise the increases available to the State would not be paid. These stronger reporting frameworks built on the trend from the previous agreements and responded to a critical Auditor-General's report that concluded that the Commonwealth did not have all the performance information required to administer the Commonwealth funding allocated under the agreements [8].

## The 'negotiation' processes

Why were the processes so acrimonious and what shaped the Agreement outcome? To some extent the shape of the 2003–08 Agreement negotiations was inevitable. The political context, where all state and territory governments were of the opposite political colour from the Commonwealth, meant that harmonious negotiations were probably never seriously in contemplation. Commonwealth governments of both persuasions have tightened up accountability on states with successive Agreements and so tighter control was also probably inevitable. Two important political choices did exacerbate the tensions and inflamed the processes.

First, the 2003–08 framework was more parsimonious than predecessor Agreements. As mentioned above, this represented a saving to the Commonwealth of about $1 billion on the Forward Estimates. A contemporary political issue was the decline in bulk billing. The Commonwealth's response to this involved an injection of around $1 billion. The link between the two policy debates within the Health portfolio is clear. Cabinet probably judged the political costs of finding a $1 billion saving from the states as low, as state premiers complaining about Commonwealth cuts and meanness is a regular part of the political landscape. Further, States would probably have criticised the Commonwealth position regardless of the offer made.

The second choice that shaped the process was the Commonwealth's intransigence after the drafts were released. The Commonwealth's position here may have been based on a recognition that, eventually, all the states would have to sign the Agreements as they were politically committed to Medicare and free access at point of admission to hospitals, and that the states could not afford to suffer the cash flow consequences announced by the Commonwealth if the Agreements weren't signed by their deadline. The Prime Minister probably took a strong hand in this decision and left no room for his Health Minister to manoeuvre. The Minister's failure to attend meetings exacerbated an already difficult situation.

A positive of the process was the extensive involvement of practitioners in the lead-up to the draft Agreements through the Reference Groups. Commonwealth-state negotiations had hitherto been an arcane process involving bureaucratic insiders. This widening of participation was welcomed by those involved and has set a precedent for future negotiations.

## Prospects for Reform

The 2003–08 Agreements commit the Commonwealth and states to work towards reform in a number of areas including the interface between hospitals, primary care, and aged care; continuity of care particularly in cancer care and mental health services; and continued work on pharmaceuticals reform. A subtle shift from the predecessor Agreement model is the more sceptical and parsimonious approach to the potential for health care reform. Despite the aspirations implicit in establishing the nine reference groups, the language of the 2003–08 Agreements reflects a much more hard-nosed approach to reform with a strong emphasis on efficiency. This approach is most clearly articulated in Clause 18: "The Commonwealth considers that for its part such reforms can take place within existing funding parameters".

Although predecessor Agreements also made provision for reform to Commonwealth/State relations, the progress in designing and implementing reform has not lived up to expectations. The most important shift that occurred during the course of the 1998–2003 Agreements was the rationalisation of hospital provision of outpatient pharmacy services, a long overdue response to a significant frictional issue in Commonwealth/States relations [9,10].

It is unclear whether the dynamic, facilitatory aspects of the 2003–08 Agreements will lead to any reform, especially given the acrimonious exchanges prior to signature of the Agreements. However it is important to note that, with a Federal election due at the end of 2004, there is a possibility that a Labor Government will be administering the remainder of the 2003–08 Australian Health Care Agreement. A new Government may be more committed to reforming and strengthening Medicare. However, despite the fact that a Labor would then hold political office throughout Australia, at all levels, this would not necessarily presage a more *laissez faire *attitude by a federal government to its state politically-allied counterparts. A Commonwealth Labor government would be just as keen as its Liberal predecessor to ensure that states are held accountable for maintaining spending and access.

## Competing interests

The author(s) declare that they have no competing interests.
